# Implementation of orbitrap mass spectrometry for improved GC-MS target analysis in lithium ion battery electrolytes

**DOI:** 10.1016/j.mex.2022.101621

**Published:** 2022-01-14

**Authors:** Christoph Peschel, Fabian Horsthemke, Martin Winter, Sascha Nowak

**Affiliations:** a University of Münster, MEET Battery Research Center, Corrensstraße 46, 48149 Münster, Germany; b Helmholtz-Institute Münster, IEK-12, Forschungszentrum Jülich, Corrensstraße 46, 48149 Münster, Germany

**Keywords:** GC-HRMS, Orbitrap mass spectrometry, Target analysis, Lithium ion battery electrolytes, BEC, *n*-butyl ethyl carbonate, BMC, *n*-butyl methyl carbonate, EI, electron ionization, EIC, extracted ion chromatogram, EPC, ethyl propyl carbonate, FWHM, full width at half maximum, GC-MS, gas chromatography – mass spectrometry, HRMS, high resolution mass spectrometry, LIB, lithium ion battery, MPC, methyl propyl carbonate, *s*BEC, *sec*-butyl ethyl carbonate, *s*BMC, *sec*-butyl methyl carbonate, SPME, solid phase microextraction, SQ, single quadrupole

## Abstract

The implementation of orbitrap mass spectrometry for target analysis of volatile species in aged lithium-ion batteries was performed in a case study on butyl carbonates. In comparison to previously applied single quadrupole-based methods, major improvements were obtained.

• Sensitivity was improved by effectively background free extracted ion chromatograms of identified marker fragment ions.
• Typical isobaric interferences of typical carbonate fragment ions e.g. caused by column bleeding were identified and false positive identification avoided.
• Analysis of isotope labeled electrolytes was optimized regarding mass spectrometric data reliability with mass accuracies <0.5 ppm and mass resolutions >100,000.

## Graphical abstract

**Figure:**
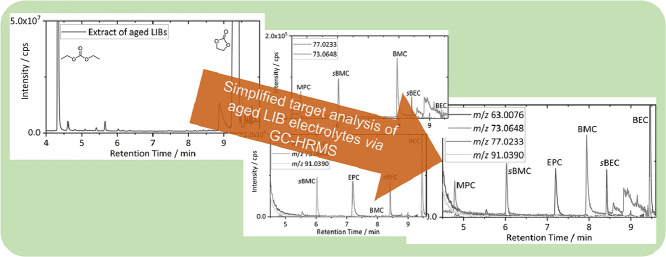

Specifications table

**Table:** 

Subject Area:	Energy
More specific subject area:	Lithium ion battery electrolyte degradation and aging
Method name:	Target analysis *via* GC-HRMS of lithium ion battery electrolyte decomposition
Name and reference of original method:	GC-MS analysis of lithium ion battery electrolyte decomposition [1,2]
Resource availability:	*N.A.*

## Method details

### Method background

Gas chromatography – mass spectrometry (GC-MS)-based analysis of volatile species in lithium ion battery (LIB) electrolytes is well established. Separation of applied organic carbonate solvent molecules and aging caused decomposition species is usually obtained on nonpolar columns with moderate temperature programs. Database comparisons of electron ionization (EI) mass spectra obtained with single quadrupole (SQ) instruments enable fast screening on applied components as well as identification of unknown species. However, in terms of peak overlapping and/or low intensities, that exclude background subtraction of mass spectra, SQ instruments with low mass resolution are stretched to their limits [3].

With ongoing research on LIB electrolyte degradation phenomena, identification of formed species was performed in low ppm levels. Within this concentration range, especially analysis of carbonate-based structures with unfavorable EI ionization properties was solely obtained after preconcentration or with low dilutions and split conditions. However, these harsh dilution parameters led to damage of the GC system, due to unavoidable LiPF_6_ salt injections [3]. To improve the analysis of LIB decomposition species, high resolution (HR)MS detection after GC separation is inevitable. Especially modern Orbitrap-based MS systems provide sub-ppm mass accuracies and mass resolution capabilities > 100,000 to simplify molecular formula prediction based on the mass defects of measured ions [4,5]. Moreover, based on extracted ion chromatograms (EICs) with effectively no background noise, sensitivity and selectivity of target analysis can be improved.

Recently, ^13^C carbon atom labeling was introduced to trace carbon atoms of electrolyte solvent molecules in observed decomposition species [6,7]. As a conclusion of these experiments, complex reaction mechanisms including labeled and unlabeled electrolyte components form the analyzed degradation products and the contained ^13^C proportions can vary. Therefore, mass spectra became very complex. Especially for carbonate-based structures with manifold fragmentation, different labeled and unlabeled fragment ions with the same nominal mass overlap. These isobaric interferences complicate isotope labeling identification. Moreover, for SQ-MS systems identification of isotope labeled fragment ions is solely based on observable mass shifts compared to unlabeled reference samples and final experimental prove is pending.

In this method report we introduce GC-Orbitrap HRMS analysis (hereinafter referred to as GC-HRMS) in the area of LIB electrolyte aging investigations. Evidence of the enhanced method capabilities was exemplarily performed on butyl carbonates [2]. 4 different species - namely *n*-butyl methyl carbonate (BMC), *sec*-butyl methyl carbonate (*s*BMC), *sec*-butyl ethyl carbonate (*s*BEC) and *n*-butyl ethyl carbonate (BEC) were analyzed *via* GC-HRMS. Thereby, simplified and more reliable identification *via* marker fragments was enabled. Moreover, proposed labeling from recent SQ-MS-based studies was confirmed on maximized experimental MS data certainty.

## Experimental details

### Analyzed electrolyte samples

Improved target analysis is shown for an extract obtained from unknown shredded LIB material *via* dichloromethane extraction (6.5 g material, 5 mL dichloromethane, intensive shaking) and subsequent filtration with a syringe filter (22 µm). The same sample was used for GC-HRMS as well as for comparative GC-SQ-MS experiments.

For detailed information on isotope labeling of LIB electrolytes and sample generation *via* electrochemical aging of the respective labeled and unlabeled electrolytes the reader is kindly referred to previous studies focusing on interpretation of observed electrolyte degradation phenomena rather than possible analytical method optimizations [6,7]. For method validation, the sample “El_DMC, lab_” was re-examined *via* SPME-GC-HRMS [7].

### GC-HRMS method parameters

The described GC-MS method is based on SQ-MS experiments described by Grützke et al. [1] (liquid injection) and Horsthemke et al. [2] (preconcentration).

GC-HRMS investigations were performed on a Q Exactive GC Orbitrap GC-MS/MS system with a TRACE 1310 GC and a TriPlus RSH autosampler (all Thermo Fisher Scientific, USA). The Q Exactive GC setup control was carried out by means of Xcalibur 4.2 SP1 (Thermo Fisher).

The injection port was operated at 250°C in split mode with ratios of 1:100 and 1:10 for liquid injection and solid phase microextraction (SPME), respectively. Liquid injection was performed with an injection volume of 1 µL *via* a 10 µL syringe. SPME preconcentration was performed manually for 10 min at room temperature from a 20 mL headspace vial. Subsequent desorption occurred at 250 °C for 60 s in the GC injector. Prior to each sample measurement, the SPME fiber was conditioned for 300 s at 260 °C and a blank of ambient air was measured to exclude carry over effects.

GC separation was performed on a nonpolar Supelco SLB-5ms (30 m × 0.25 mm; 0.25 μm) column (Sigma Aldrich, Germany). Helium 6.0 (Westfalen, Germany) was used as carrier gas in constant column flow mode, set at 1.16 mL min^−1^. The temperature program started at 40 °C, which was held for 1 min followed by ramps of 3 °C min^−1^ up to 60 °C and 30 °C up to 260 °C. The final temperature was held for 2 min. Transferlines to the Q Exactive system were operated at 250 °C.

Ionization was carried out by EI mode. Ion source parameters were set based on the current auto tune results for TIC tuning with 70 eV electron energy. The MS was operated in Full MS mode for positive polarity with a set resolution of 60,000 full width at half maximum (FWHM) at *m*/*z* 200. The automatic gain control target was set at 1*10^6^ and the maximum injection time was set to auto. The mass range was set to *m*/*z* 35–500 and mass spectra were recorded in profile type. Depending on the measured sample, the filament and MS detection started delayed (solvent cut) due to highly concentrated solvent molecules with low retention times.

Data analysis like chromatogram extraction and mass peak resolution determination was performed with FreeStyle 1.5 (Thermo Fisher). EICs were extracted from Full MS experiments based on measured *m*/*z* values with a mass window of 5 ppm. Chromatograms and spectra were plotted with OriginPro 2019b (OriginLab, USA).

A summarizing workflow with additional comments to reproduce the described LIB electrolyte analysis is shown in Fig. S3. Moreover, the limits of detection for the overall method were determined for the most prominent carbonate decomposition species dimethyl-2,5-dioxahexane carboxylate (DMDOHC) and diethyl-2,5-dioxahexane carboxylate (DEDOHC) (Figure S4/S5).

## Method implementation and validation

### Improvements of sensitivity and reliability of targeted GC-MS analysis

**Fig. 1:**
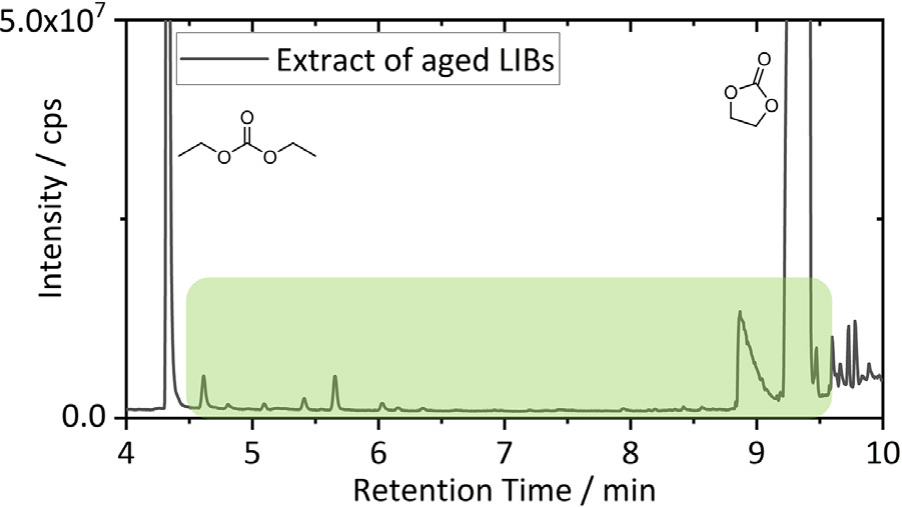
GC-HRMS chromatogram of organic residues extracted form aged LIBs. The retention window of targeted carbonate species is marked in green.(For interpretation of the references to color in this figure legend, the reader is referred to the web version of this article.)

Fig. 1 shows the retention time of interest (4.5–9.6 min) of a Full MS GC-HRMS chromatogram of extracted organic compounds from LIBs. In the GC-HRMS chromatogram after liquid injection small peaks were found in the contemplated retention window, however, fast identification based on retention behavior was inconclusive. Previously, butyl carbonates were solely identified after SPME preconcentration due to sensitivity issues [2]. For method comparison, the same sample was also measured *via* GC-SQ-MS with the same injection volume, split ratio and MS parameters based on auto tune results. Despite extraction of marker fragments, the targeted molecules could not be detected *via* liquid injection (Fig. S1/S2).

**Fig. 2:**
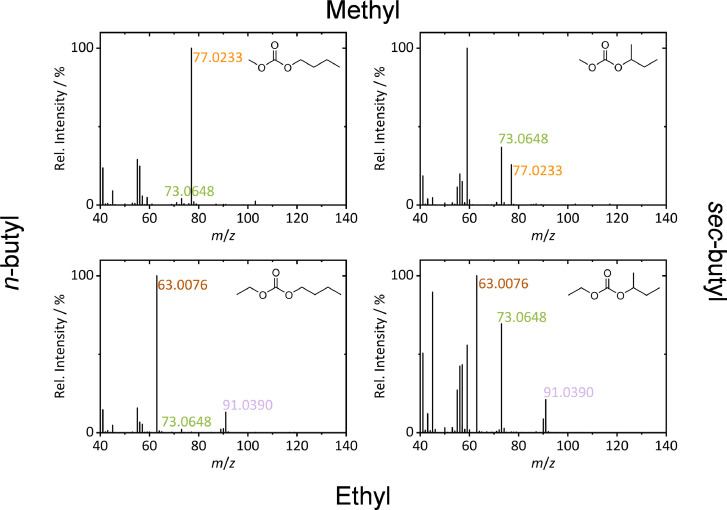
GC-HRMS mass spectra of the four target molecules. Characteristic fragment ions for methyl carbonates (orange), ethyl carbonates (brown, violet) and butoxy (green) moieties are marked.(For interpretation of the references to color in this figure legend, the reader is referred to the web version of this article.)

To improve target analysis by means of GC-HRMS, EICs of accurate *m*/z values of characteristic target ions are helpful for enhanced selectivity. To identify these characteristic fragment ions, the HRMS mass spectra of previously synthesized standards [2] of the 4 target molecules are depicted in Fig. 2.

**Fig. 3:**
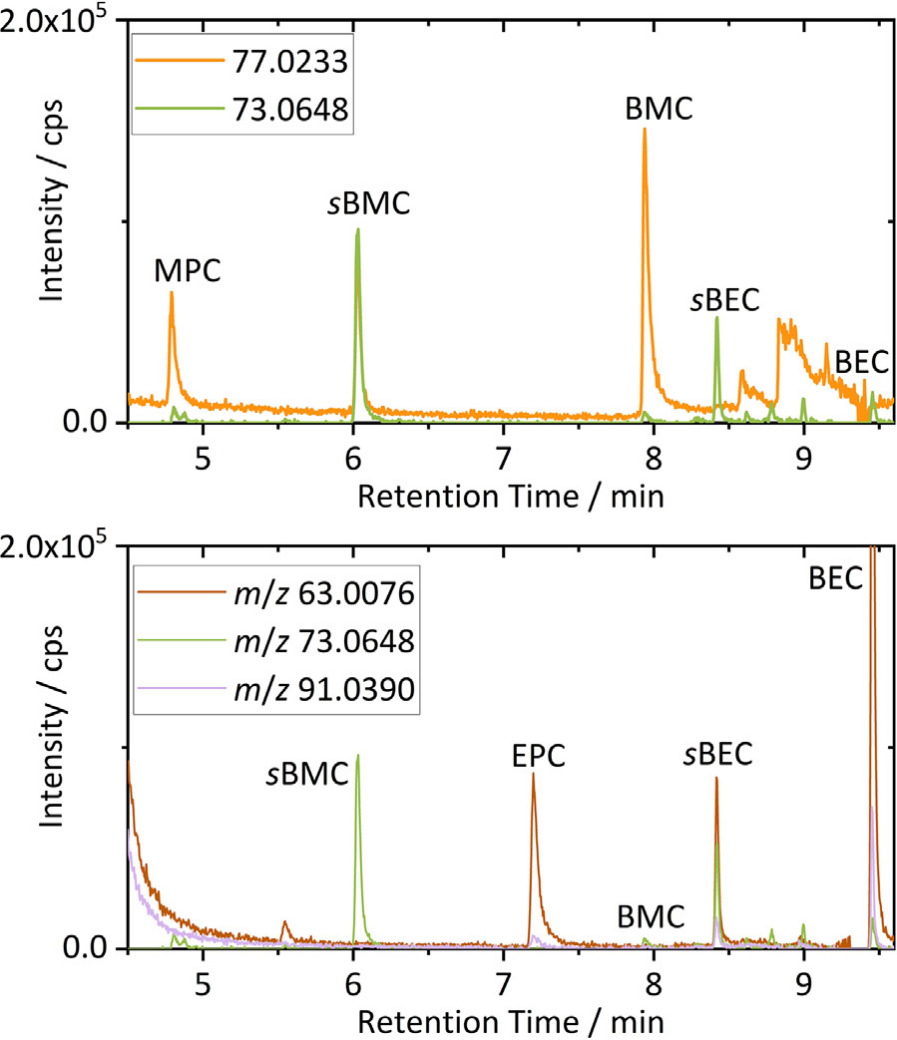
Overlays of GC-HRMS EICs extracted from the chromatogram shown in Fig. 1. For identification of butyl methyl carbonates (top) and butyl ethyl carbonates (bottom) extracted *m*/*z* values were chosen based on characteristic fragment ions with a mass window of 5 ppm. Further literature known decomposition species are also marked [2]. Color code is applied according to Fig. 2.

By comparison of the obtained mass spectra and structure-based considerations on fragmentation behavior, marker fragments for substructures were identified. Methyl butyl carbonates were identified *via* the methyl carbonate fragment ion with *m*/*z* 77.0233 (C_2_H_5_O_3_). For ethyl butyl carbonates, fragment ions with *m*/*z* 63.0076 (CH_3_O_3_) and 91.0390 (C_3_H_7_O_3_) were formed, not observed in the methyl species (Fig. 2). Further, the butoxy moieties can be screened *via* the formed fragment ion with *m*/*z* 73.0648 (C_4_H_9_O). The butoxy fragment is preferably formed for *sec*-butyl carbonates. However, in combination with the respective methyl or ethyl carbonate marker fragments BMC and BEC were also identified. The improved identification of butyl carbonates as LIB aging products *via* EICs of the exact masses of marker fragments for methyl and butoxy as well as for ethyl and butoxy moieties is depicted in Fig. 3.

In addition to the exemplarily discussed and targeted butyl carbonates, further methyl and ethyl carbonate-based structures were identified in the considered retention time window. These were also identified in previous studies as methyl propyl carbonate (MPC) and ethyl propyl carbonate (EPC) [2]. On the one hand, this findings illustrate the applicability of carbonate marker fragment ions not only for targeted analysis of the aimed species, but also for screening of unknown mixtures. Marker fragment ions simplify identification of further species that could contain similar substructures. On the other hand, the fast identification of further species demonstrates, that more general target lists for a majority of literature known carbonate-based decomposition species are hardly achievable. Due to structural similarities of species containing mainly carbonate, glycol and alkyl moieties, fragmentation of larger (oligomeric) species results in the same “marker” fragment ions. Furthermore, EI fragmentation pathways can be very complex and uncharacteristic towards molecule ion structures e.g. due to rearrangements. Nevertheless, screening with EICs of marker fragment ions can also be considered for fast sample screening in non-targeted approaches.

**Fig. 4:**
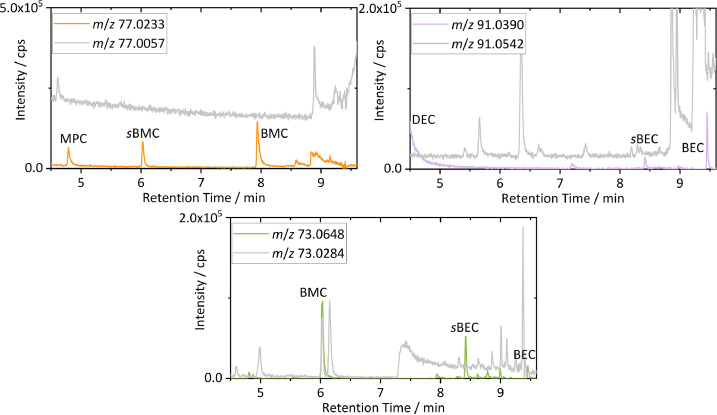
Overlays of GC-HRMS EICs extracted from the chromatogram shown in Fig. 1. For illustration of HRMS capabilities for interference free EICs, overlays with background signals with identical nominal masses are shown. Color code is applied according to Fig. 2 with background interferences drawn in gray.

A further advantage of HRMS is the identification and exclusion of possibly interfering isobaric background noise. Especially for targeted analysis on low concentrated analytes, interferences can lead to false positive identification. Comparisons of EICs of exemplarily observed isobaric interferences (e.g., *m*/*z* 73.0284, 77.0057 and 91.0542) on previously discussed nominal masses of respective target fragments are depicted in Fig. 4.

With the Orbitrap MS resolution set at 60,000 (FWHM) at *m/z* 200 all major interferences on investigated species were avoided and effectively background free EICs enabled. For example, the high background of *m*/*z* 91.0542 caused by the tropylium cation (C_7_H_7_), which is highly stable and frequently encountered as fragment of various aromatic structures with benzylic moieties, was identified and avoided.

### Improved reliability for analysis of isotopic constitution

Isotope labeling of one solvent molecule from a binary mixture resulted in varying labeling distributions in observed decomposition species [6,7]. The analysis of these decomposition species *via* SPME-GC-SQ-MS suffered from further interferences of different labeled and unlabeled fragment ions with the same nominal mass. The implementation of GC-HRMS provided molecular formula prediction with exact isotopic constitution for these fragment ions. In the previous study, labeling was concluded indirectly *via* mass shifts of proposed fragment ions. However, concrete experimental prove is pending.

**Fig. 5:**
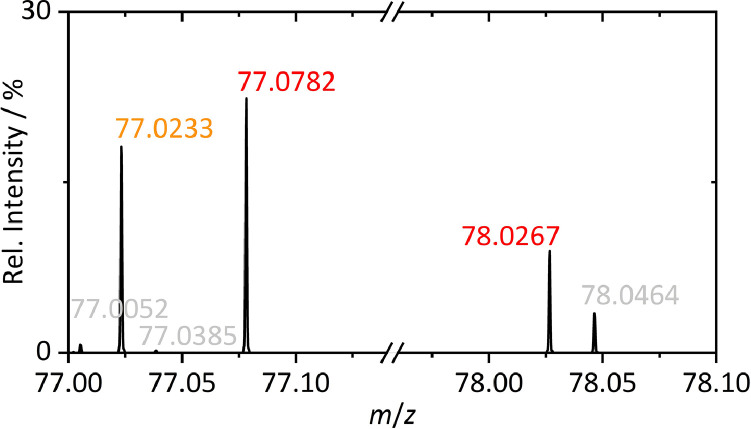
Magnified SPME-GC-HRMS profile type mass spectra of sBMC obtained from a partially ^13^C isotope labeled LIB electrolyte [7]. ^13^C atom(s) containing fragment ions are marked in red. Background noise (gray) and the methyl carbonate fragment (orange) are marked according to previous color code.(For interpretation of the references to color in this figure legend, the reader is referred to the web version of this article.)

Again, the capabilities of GC-HRMS for analysis of isotope labeling of LIB decomposition species are illustrated for butyl carbonates - more precisely for *s*BMC and the identification of the fourfold ^13^C labeled butoxy moiety (^13^C_4_H_9_O; *m*/*z* 77.0782) alongside the methyl carbonate fragment with and without a ^13^C atom at the carbonyl carbon position (C^13^CH_5_O_3_: *m*/*z* 78.0267; C_2_H_5_O_3_: *m*/*z* 77.0233)). For further improved sensitivity, SPME preconcentration was applied (Fig. 5).

Mass resolution set at 60,000 (FWHM) at *m*/*z* 200 enabled mass resolutions of 115,502 and 115,202 FWHM for the observed fragment ions at *m*/*z* 77 and 78, respectively, with mass accuracies <0.5 ppm. The profile type MS data of nominal masses 77 and 78 illustrates demand and capability of resolving power for analysis of partially isotope labeled carbonates. Mass accuracy <5 ppm ensures differentiation of C_2_H_5_O_3_ and ^13^C_4_H_9_O and clear identification of C^13^CH_5_O_3_ despite background noise of C_6_H_6_ with *m*/*z* 78.0464. With effective resolving power >100,000, ^13^C carbon atom identification can be performed on maximized MS data reliability. In contrast to GC-SQ-MS direct identification of labeled species and fragments is enabled and also fragment ion intensity contribution to the nominal mass peak of 77 can be revealed.

## Conclusions

Implementation of Orbitrap MS detection into GC-MS analysis of LIB electrolytes led to improvements of sensitivity, selectivity and data reliability for target analysis. Identification of targeted species was significantly improved based on EICs of identified marker fragment ions. Thereby, mass resolution and mass accuracy capabilities of Orbitrap MS ensured effectively background free extracted masses at sub-ppm accuracy. Even high isobaric background noise of e.g. typical benzylic fragment ions did not interfere target masses.

Additionally, GC-HRMS was applied to prove mass shifts of fragment ions of previously described analytes caused by partial ^13^C labeling. More reliable identification of isotope labeling was obtained based on direct isotopic composition measurement of target fragment ions and isobaric interferences.

## Outlook on GC-HRMS for LIB electrolyte research

To further benefit from GC-HRMS capabilities also for non-target analysis, molecular ion information is essential, but not archivable for most larger carbonates-based species after EI ionization. One solution to overcome this, is the extended use of synergistic effects between strengths of GC-SQ-MS and GC-HRMS analysis. While GC-SQ-MS enables fast data evaluation *via* robust database comparisons, GC-HRMS provides more detailed information to validate molecular formulas and isotopic constitutions of observed fragments. A second option will be the implementation of softer chemical ionization for GC-HRMS to measure molecular ion adducts, since the improved selectivity *via* HRMS detection can help to overcome sensitivity drawbacks caused by chemical ionization.

Despite these remaining challenges for non-targeted analysis, the value of GC-HRMS in the area of battery research presumably will further increase with the emergence of next generation batteries. These systems contain a broader range of elements than C,H,O, P and F - e.g. sulfur and nitrogen hetero atoms - with more characteristic mass defects and natural isotope distributions [8]. For these additional analytes GC-HRMS will enable fast identification of aging products e.g. *via* targeted analysis of hetero atom containing fragment ions and simplified verification by isotopic satellites.

Supplementary material: Supplementary material with comparison measurements *via* GC-SQ-MS, the summarizing workflow, the determination of the limits of detection and the method application for carbonate trimer target analysis is attached.

## Declaration of Competing Interest

The authors declare that they have no known competing financial interests or personal relationships that could have appeared to influence the work reported in this paper.

## Appendix Supplementary materials

Image, application 1
