# Supplementary material for: Implementation of orbitrap mass spectrometry for improved GC-MS target analysis in lithium ion battery electrolytes
Source: MethodsX. 2022 Jan 14;9:101621. doi: 10.1016/j.mex.2022.101621 (PMC8790629; doi:10.1016/j.mex.2022.101621)
Supplement: Supplementary file 1 [file mmc1.docx]

**Implementation of Orbitrap Mass Spectrometry for Improved GC-MS Target Analysis in Lithium-Ion Battery Electrolytes**

Christoph Peschel^a^, Fabian Horsthemke^a^, Martin Winter^a,b^ and Sascha Nowak^a^*^*^*

*^a^ University of Münster, MEET Battery Research Center, Corrensstraße 46,
48149 Münster, Germany*

*^b^ Helmholtz-Institute Münster, IEK-12, Forschungszentrum Jülich, Corrensstraße 46,
48149 Münster, Germany*

**Corresponding author: e-mail address: sascha.nowak@uni-muenster.de*

# GC-SQ-MS experiments

GC-SQ-MS with liquid injection experiments were executed on a Shimadzu GCMS-QP2010 Ultra with assembled AOC-5000 Plus autosampler and a nonpolar Supelco SLB^®^-5ms (30 m×0.25 mm. 0.25 µm; Sigma Aldrich, Germany) column. For best method comparison, further parameters were applied according to the GC-HRMS method, based on the method described by *Grützke et al.*^[1]^


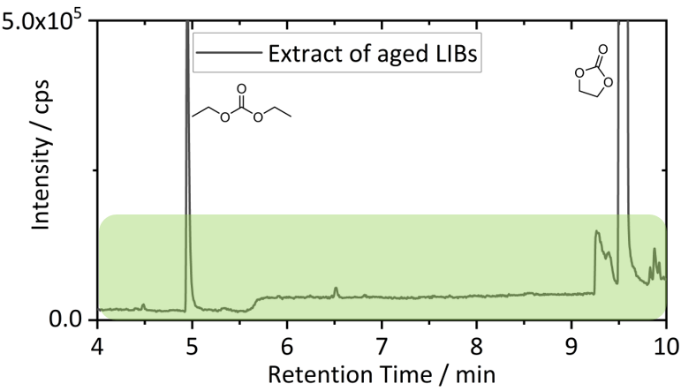


Figure S1: GC-HRMS chromatogram of organic residues extracted form aged LIBs. The retention window of targeted carbonate species is marked in green.


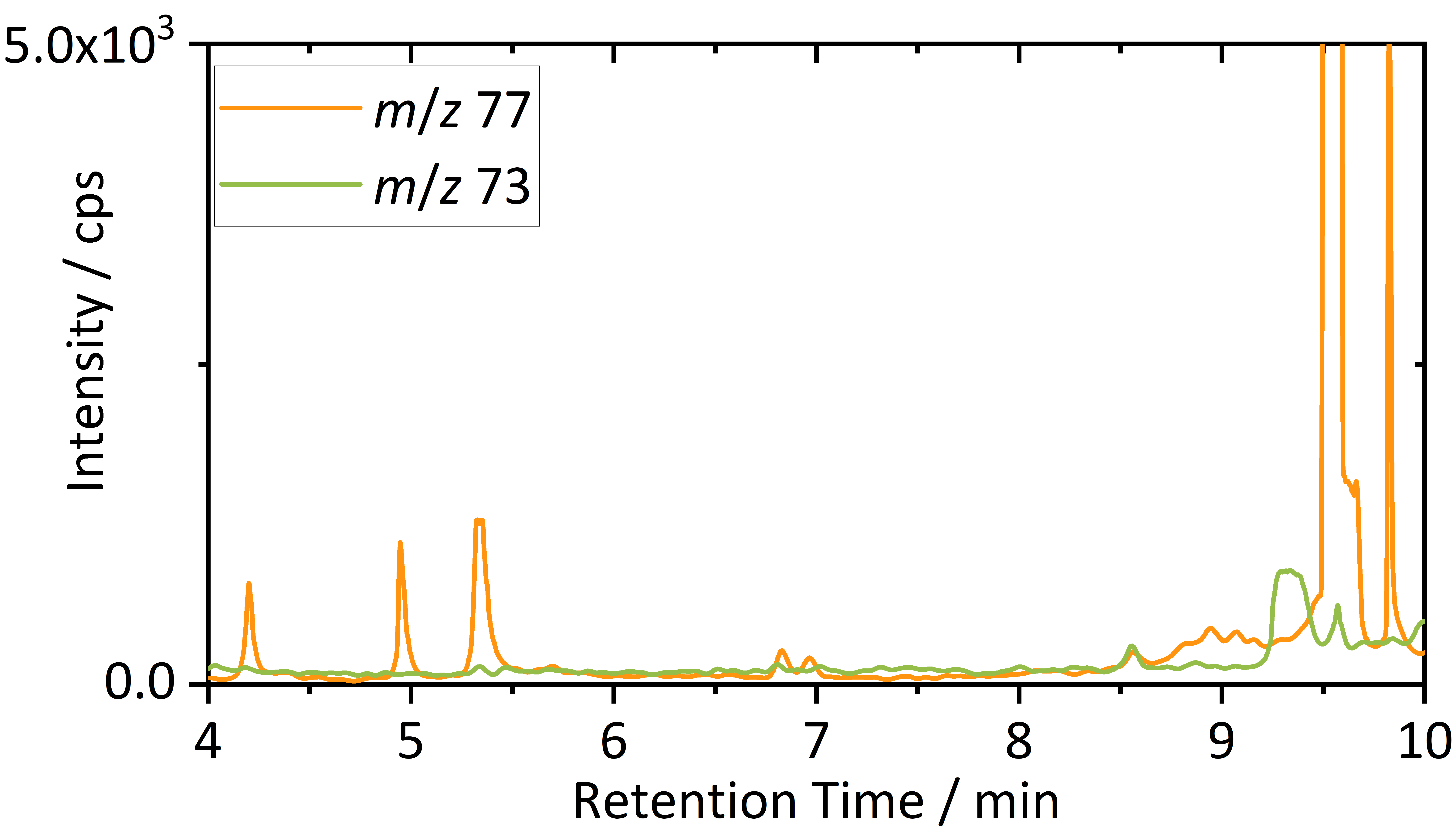


Figure S2: GC-SQ-MS EICs of (s)BMC marker fragments. Color code is applied according to Figure 2.

# Workflow

The summarizing workflow is depicted in Figure S3.


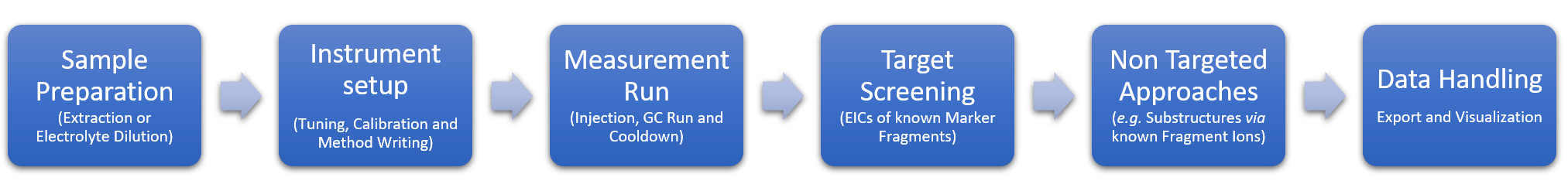


Figure S3: Summarizing workflow for LIB electrolyte analysis.

Starting with the sample preparation harmful LiPF_6_ injection into the GC has to be avoided. Not only to lower column bleeding, but also to protect the injection part, precipitation in nonpolar solvents or headspace-(SPME) analysis is advisable. Moreover, to prevent ongoing electrolyte decomposition, moisture and thermal stress should be avoided during sample preparation. Depending on the LIB constitution, electrolyte recovery and subsequent dilution can take up to a whole workday. (Excluding conducting salt precipitation overnight)

The instrument setup was already described in detail. A daily calibration of the orbitrap mass analyzer is mandatory to ensure mass resolution and accuracy as reported. From starting the instrument, the instrument setup should take no more than 15 minutes. After setting up the batch, each sample measurement takes around half of an hour including injection and GC cooldown.

Afterwards, data evaluation takes most of the time. Targeted approaches, as reported in this study, can be completed in a couple of minutes, however, non targeted approaches are more challenging and time consuming.

**Limits of detection**

Due to lack of commercially available standards of the discussed butyl carbonates, the limits of detection of the overall method were determined for the most prominent carbonate decomposition species. Dimethyl-2,5-dioxahexane carboxylate (DMDOHC, > 98 %) and diethyl-2,5-dioxahexane carboxylate (DEDOHC, > 98%) were obtained from ABCR (Germany) and diluted gravimetrically in 10 samples ranging equidistantly from 0.1 to 1 ppm. All samples were measured in threefold determination and the limits of detection were calculated based on the obtained calibration curve. The considered EICs were chosen based on the most prominent fragment ions. (Figure S4).


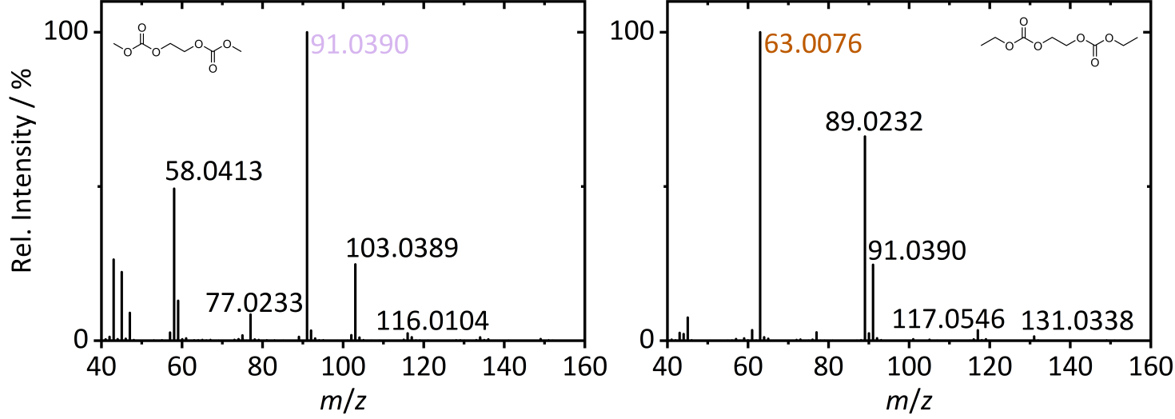


Figure S4: GC-HRMS mass spectra of the considered dimers (DMDOHC, left; DEDOHC, right). Characteristic fragment ions for LOD determination via EICs are marked.

The calculated LODs of DMDOHC (< 500 ppb) and DEDOHC (< 200 ppb) illustrate ionization efficiency dependent LODs of the method. However, analysis in the sub-ppm concentration range was ensured, despite the moderate split of 1:100, to minimize column bleeding by injection of conducting salt residues. To visualize sub-ppm analysis of the carbonate oligomers, the threefold determination of a sample with gravimetrically spiked 190 ppb DEDOHC is exemplarily shown in Figure S5.

*
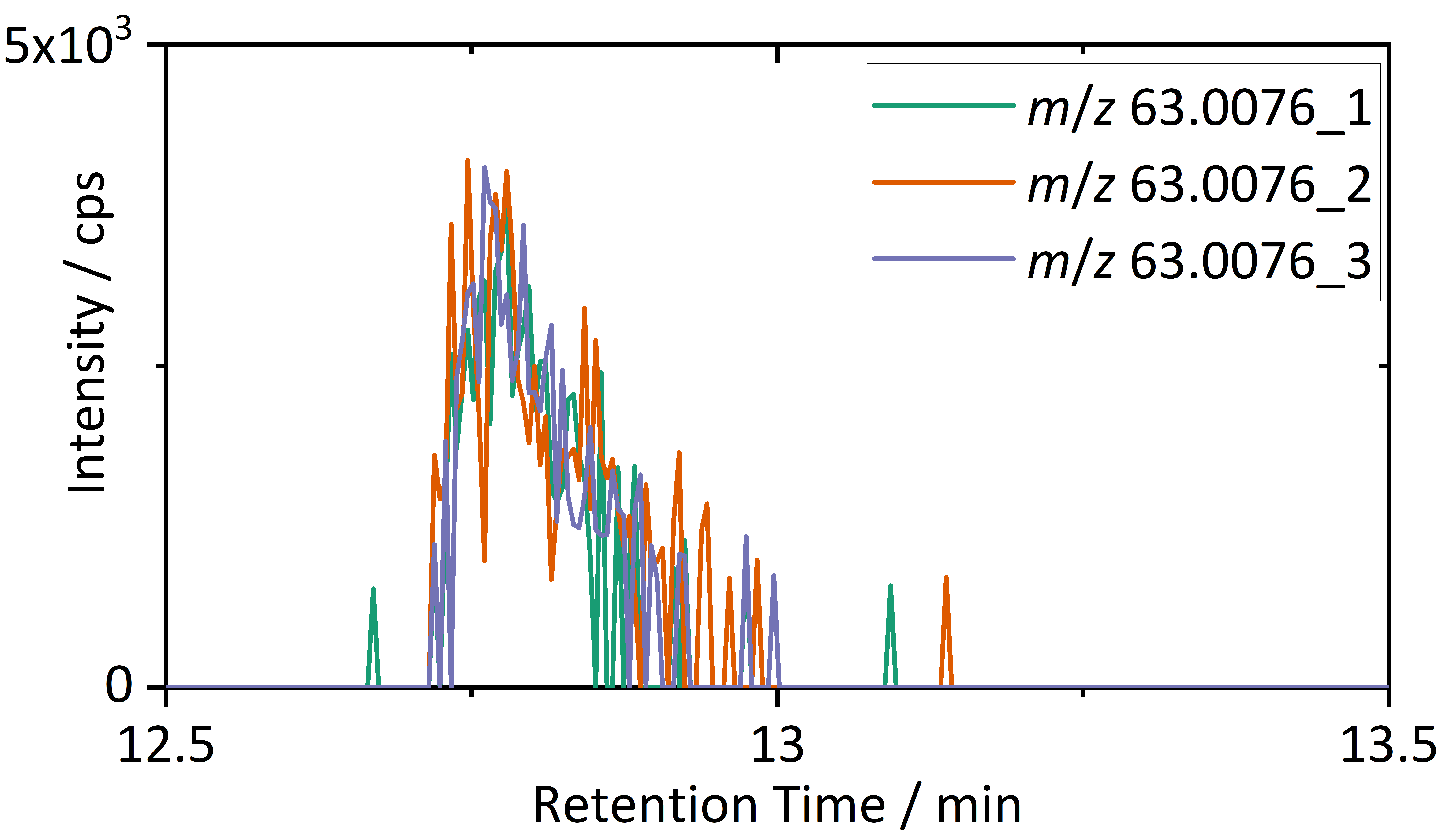
*

Figure S5: GC-HRMS EICs of a DEDOHC standard (190 ppb) within the LODs of the described method.

**Carbonate oligomer target analysis**

In addition to DEDOHC, screening with defined carbonate marker fragments also enables identification of carbonate trimers (M = 266 - 294 g mol^–1^) at higher retention times. Carbonate trimers were previously described with LC-MS, but improved LIB electrolyte analysis *via* GC-HRMS also enables identification by GC analysis. The identification of this species is exemplarily shown for an EMC-based electrolyte after cell formation and was validated with softer ionization *via* GC-APCI-Q-TOF-MS.^[2]^ (Figure S6)


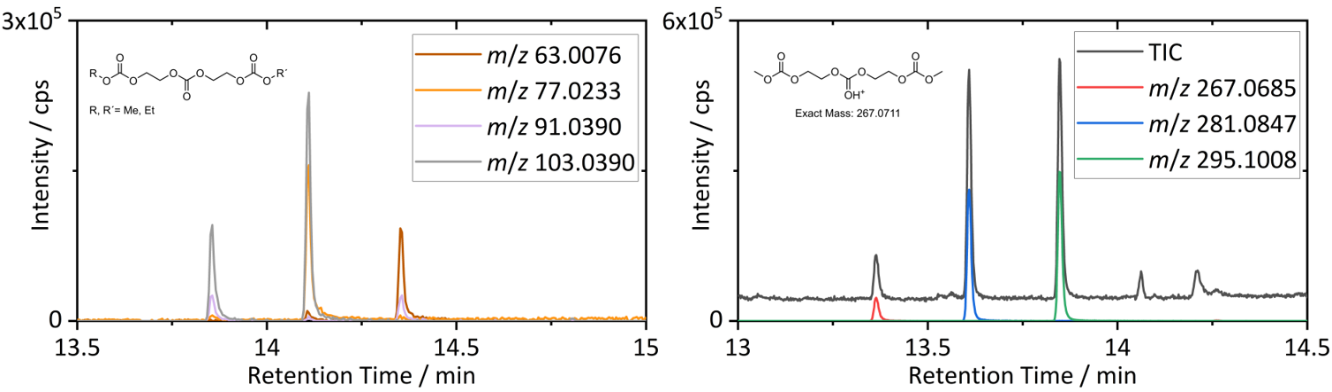


Figure S6: Identification of carbonate trimers via EICs of characteristic fragment ions (left) and via soft chemical ionization (right). Shifted retention times are caused by different GC-HRMS instrumentation.

# References

[1] M. Grützke, X. Mönnighoff, F. Horsthemke, V. Kraft, M. Winter, S. Nowak, *RSC Adv.* **2015**, *5*, 43209–43217.

[2] Y. Preibisch, C. Peschel, J. F. Dohmann, M. Winter, S. Nowak, *J. Electrochem. Soc.* **2021**, *168*, 026501.
